# Evaluation of Sequence Features from Intrinsically Disordered Regions for the Estimation of Protein Function

**DOI:** 10.1371/journal.pone.0089890

**Published:** 2014-02-24

**Authors:** Alok Sharma, Abdollah Dehzangi, James Lyons, Seiya Imoto, Satoru Miyano, Kenta Nakai, Ashwini Patil

**Affiliations:** 1 School of Engineering and Physics, The University of the South Pacific, Suva, Fiji; 2 Institute for Integrated and Intelligent Systems, Griffith University, Brisbane, Australia; 3 National Information and Communication Technology Australia (NICTA), Brisbane, Australia; 4 School of Engineering, Griffith University, Brisbane, Australia; 5 Human Genome Center, The Institute of Medical Science, The University of Tokyo, Tokyo, Japan; Weizmann Institute of Science, Israel

## Abstract

With the exponential increase in the number of sequenced organisms, automated annotation of proteins is becoming increasingly important. Intrinsically disordered regions are known to play a significant role in protein function. Despite their abundance, especially in eukaryotes, they are rarely used to inform function prediction systems. In this study, we extracted seven sequence features in intrinsically disordered regions and developed a scheme to use them to predict Gene Ontology Slim terms associated with proteins. We evaluated the function prediction performance of each feature. Our results indicate that the residue composition based features have the highest precision while bigram probabilities, based on sequence profiles of intrinsically disordered regions obtained from PSIBlast, have the highest recall. Amino acid bigrams and features based on secondary structure show an intermediate level of precision and recall. Almost all features showed a high prediction performance for GO Slim terms related to extracellular matrix, nucleus, RNA and DNA binding. However, feature performance varied significantly for different GO Slim terms emphasizing the need for a unique classifier optimized for the prediction of each functional term. These findings provide a first comprehensive and quantitative evaluation of sequence features in intrinsically disordered regions and will help in the development of a more informative protein function predictor.

## Introduction

Computational protein annotation methods are gaining increasing importance with the sequencing of a large number of organisms. Function prediction is done by assigning Gene Ontology terms [1] to proteins. These methods use different characteristics of proteins such as their sequence, presence of distant homologs, predicted secondary structure, binding partners, coexpressed genes, etc. to predict their function [2]. However, very few methods directly use the properties of intrinsically disordered regions (IDRs) in function prediction. Intrinsically disordered regions are known for their flexibility and binding promiscuity, and are important functional regions in proteins [3]. IDRs have been found to be enriched in hub proteins and are often associated with specific ordered domains [4], [5]. They have also been found to contain functional sequence motifs [6], [7]. IDRs have been known to directly affect protein function based on their characteristics. An early study identified different types, or flavors, of disorder enriched for specific functions [8]. Additionally, it has been shown that the chemical composition of IDRs can be directly associated with the functions of their parent proteins [9]. Thus, using information from these regions may help improve function prediction of proteins. However, their lack of stable tertiary structure, low sequence complexity and poor sequence conservation make them difficult to use for function prediction with existing methods, which are more suited for ordered proteins. Jones and colleagues have used the length of IDRs along with their location within the protein to identify associated functional terms [10], [11]. We have previously used amino acid composition of IDRs to assign functional terms to proteins [12]. Though these methods have moderate success in assigning functional terms, systematic studies extracting and assessing feature vectors from IDRs have so far been lacking.

In this study, we attempted to predict protein function using the sequence features of each IDR present within the protein. We extracted several sequence features from IDRs within proteins and tested their ability to assign the appropriate GO Slim terms to the protein. The sequence features included those that were based on residue frequency, predicted secondary structure, as well as those that depended on sequence profiles obtained using remote homologs. Our results indicate that while features based on residue composition and secondary structure have higher precision, those based on PSIBlast profiles [13] have the highest recall (sensitivity). Additionally, IDR features vary in their ability to classify proteins by function with some features performing better than others for specific functional terms.

## Methods

### Dataset

IDRs predicted using DisoPred2 [14] in human proteins were obtained from Moesa et al. [9]. 11329 IDRs from 6751 proteins annotated with 130 GO Slim terms were used in the analysis (Table S1). Each protein had between 4–7 orthologs among chimp, dog, mouse, rat, fly, worm and yeast (Table S2). This dataset has been previously used to show the relationship between the chemical composition of IDRs and the functions associated with the IDR-containing protein. We assumed that all IDRs within a protein contribute towards its overall function. Hence, each IDR was assigned the GO Slim terms of its parent protein. GO Slim terms were used instead of GO Molecular Function or GO Biological Process terms since they help combine several specific terms into a single class reducing the number of classifiers necessary and increasing the feature vectors that can be used for their training. The number of GO Slim terms assigned to a protein varied from 1 to 43 with 74% of the proteins having 10 or fewer terms (Figure S1).

### Sequence Features

We used the following sequence features to describe each IDR within a protein (Table 1). Each feature consisted of a vector of values used to represent the IDR sequence.

**Table 1:** Features extracted from sequences of intrinsically disordered regions.

Feature	Reference	Dimensions
Chemical composition	Moesa et al., 2012 [9]	5
Amino acid composition	Patil et al., 2012 [12]	20
Composition+Dubchak features	Ding and Dubchak, 2001 [15]	125
Occurrence+Dubchak features	Taguchi and Gromiha, 2007 [20]; Ding and Dubchak, 2001 [15]	125
Sequence bigrams	Ghanty and Pal, 2009 [17]	400
Alternate bigrams	Ghanty and Pal, 2009 [17]	400
Profile bigrams	Sharma et al., 2013 [18]	400

#### 1. Chemical composition

The fraction of positively charged (Arg, Lys), negatively charged (Asp, Glu), polar (Ser, Thr, Asn, Gln, Tyr, Cys), hydrophobic (Ala, Val, Leu, Ile, Met, Phe, Trp) and special (Pro, Gly) residues in the IDR [9].

#### 2. Amino acid composition

The fraction of each of the 20 amino acids within the IDR [12].

#### 3. Composition+Dubchak features

Dubchak features were previously used to predict protein folds [15]. They include amino acid composition, hydrophobicity, predicted secondary structure, polarity, polarizability and normalized van der Waals volume. The size of this feature vector is 125.

#### 4. Occurrence+Dubchak features

The amino acid occurrence i.e. the un-normalized number of each amino acid, is used instead of amino acid composition [16]. Dubchak features other than amino acid composition are also used. The size of feature vector of this feature is 125.

#### 5. Residue bigram probabilities

This feature incorporates the probabilities of the occurrence of all amino acid dimer pairs in the IDR [17]. This is a 400 dimensional feature vector.

#### 6. Alternate bigram probabilities

This feature vector consists of the probabilities of occurrence of all possible pairs of amino acids that are separated by one residue in the IDR sequence [17]. This is also a 400 dimensional feature vector.

#### 7. Position specific scoring matrix based bigrams (profile bigrams)

These bigrams represent the probabilities of transition from one amino acid to another as determined by the position specific scoring matrix (PSSM) obtained from PSIBlast [13]. PSIBlast was run for 3 iterations on the IDR sequence to find remote homologs in NCBI’s non redundant protein database with an e-value of 0.001. PSSM provides the substitution probability of a given amino acid based on its position along a protein sequence. An IDR sequence is represented by its PSSM, and the bigram features [18] are computed using the probability information contained in the PSSM.

Let 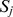 be the matrix representing the PSSM of a given IDR sequence *j*. The matrix 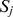 will have 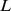 rows and 20 columns (where 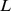 is the length of the primary sequence). Its element at 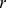
th-row and 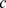
th-column is denoted by 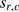 which can be interpreted as the relative probability of 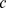
th amino acid at the 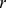
th location of the IDR sequence (with 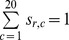 for 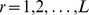). The frequency of occurrence of transition from 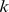
th amino acid to 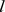
th amino acid is computed as follows
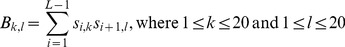
(1)


Equation 1 gives the 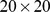 bigram features of 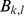. It can be interpreted in the form of a feature vector of dimension 400 as
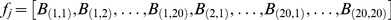
(2)


### Prediction Scheme for GO Slim Terms

**Figure 1:**
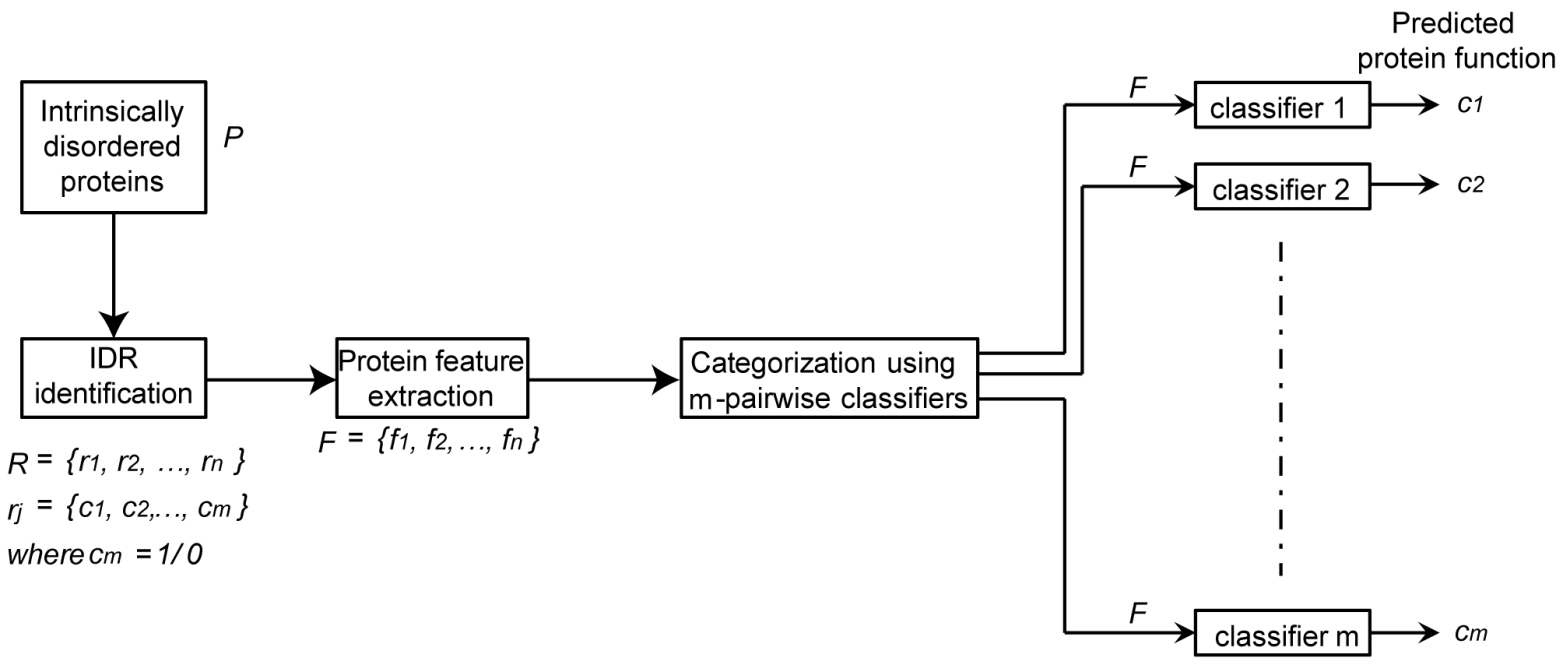
Schematic diagram of the prediction of protein function using features of intrinsically disordered regions with m-pairwise classifiers. Features *F* are extracted from proteins P that have IDRs R. Protein GO Slim terms *c_1_, c_2_…c_m_* are assigned to IDRs. A single pairwise classifier is trained for each of the *m* GO Slim terms. The classifier is used to predict a GO Slim term for a protein using features of each IDR it contains.

Figure 1 illustrates the 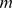-GO term prediction method used in this study. The prediction method was subdivided into two main tasks: feature extraction and classification. In the feature extraction task, we identified IDRs in each protein and computed features for IDR sequences. Let *P* denote a set of proteins containing 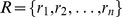 intrinsically disordered regions, where 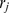 (for 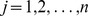) is *j*th IDR sequence. The feature vector 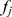 for IDR sequence 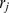 can be interpreted as
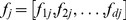
(3)where 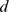 is the dimensionality or size of a feature vector 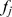. Therefore, using a feature extraction method we can extract all IDR sequences 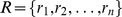 as 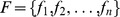.

An IDR sequence 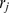 represents a set of GO Slim terms that are associated with the protein in which it is found; i.e. 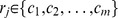, where 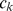 denotes GO Slim term. Immediate ancestor terms of the associated GO Slim terms were also included in the annotations of the IDR sequence to account of the parent-child relationships between the terms. The value of 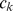 can be either 1 or 0, where ‘1′ denotes that the IDR 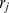 is annotated with the 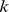
th GOSlim term and ‘0′ denotes that it is not annotated by that term. Since IDR sequences are represented by feature vectors 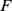, each feature vector 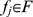 describes the GO Slim terms as given by 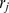.

In a classical classification problem, a feature vector belongs to only one class label. Here a feature vector belongs to multiple classes or several GO Slim terms, simultaneously. Therefore, it is imperative to break down the multiple class problem into a set of binary class classification problems. In Figure 1, 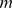-classes, or GO Slim terms, are arranged into 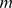 binary classes. Thus, for a particular binary class, a set of feature vectors 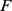 would only have a unique class label 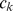 and the value of 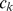 (either 1 or 0) will denote the status of 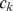 (i.e., whether a particular GOSlim term is present or absent). A classifier is then used to predict class labels of this binary class. This prediction of a feature vector is applied for all binary classes covering all GO Slim terms 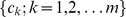. The predicted GO Slim term (i.e., terms with 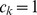) from each of the binary classifiers is collated to give the final predicted terms for a protein.

### Training and Classification

The prediction problem was divided into 130 pairwise prediction problems (one-against-others) as shown in Figure 1. Each pairwise prediction was used to classify the IDRs between a positive class and a negative class. A positive class was true if a GO term or its parent term was correctly predicted. A negative class was true if an absent GO term was classified as absent. The performance of the classifiers was evaluated using 10-fold cross-validation.

We calculated the sensitivity (recall), specificity and precision for each of the 130 classifiers for each feature using 10-fold cross-validation as
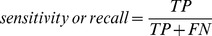
(4)




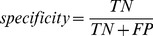
(5)




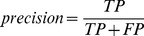
(6)where TP = true positives; i.e., number of IDRs for which the classifier correctly assigns a GO Slim term or its immediate ancetor to the protein.

FP = false positives; i.e., number of IDRs for which the classifier assigns a GO Slim term to the protein even though it is not annotated by that term.

TN = true negatives; i.e., number of IDRs for which the classifier correctly does not assign a GO Slim term to the protein.

FN = false negatives; i.e., number of IDRs for which the classifier does not assign a GO Slim term to the protein even though it is annotated with that GO term.

The sensitivity corresponds to the true positive rate of the predictor i.e. the fraction of IDRs whose features correctly predict a GO Slim term, or its ancestral term, assigned to the parent protein. The false positive rate indicates the fraction of IDRs using which the GOSlim term associated with the parent protein is incorrectly predicted. The specificity corresponds to 1– false positive rate. The precision is a measure of the fraction of correct positive predictions made. At random, a classifier has the same sensitivity (true positive rate) and 1-specificity (false positive rate). Therefore, all classifiers having a false positive rate less than the true positive rate (or specificity greater than the sensitivity) are performing better than random. The average values were calculated over all GO Slim term classifiers for each feature.

Several classifiers were tested - Naïve Bayes, kNN, AdaBoost, Bagging, Logistic regression, J48, Random Forest and SVM (See Text S1 for a brief description of the classifiers). These are utilized from Weka [19]. The Naïve Bayes classifier showed high sensitivity and predicted the largest number of GO Slim terms with higher accuracy than the other classifiers (Table S3, Figure S2). We also tested the performance of the logistic regression classifier on all the sequence features to confirm that it performed poorly compared to the Naïve Bayes classifier (Figure S3). Based on these results, we chose the Naïve Bayes classifier for further analysis.

The Naïve Bayes classifier assigned a prediction probability to each predicted class or GO Slim term. As described in a previous study [11], Mathew’s correlation coefficient (MCC) was used to assess the classification performance of each feature per GO Slim term using values as calculated above at a prediction probability of 0.5.
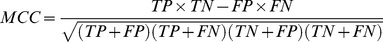
(7)


We performed the precision-recall analysis to compare the overall prediction accuracy of the feature classifiers [11]. We used 105 randomly selected IDRs as the test set, while using the remaining IDRs in the dataset as training data and predicted the GO Slim terms of the parent proteins using each feature. The precision and recall for each IDR per feature classifier was calculated at a specified prediction probability *p* as follows.

TP*r, p* = the number of known GO Slim terms of the parent protein that are predicted at a probability *p* or higher.

FP*r, p* = the number of GO Slim terms predicted for the parent protein by the IDR features at probability *p* or higher but not present in its list of known GO Slim terms.

FN*r, p* = the number of known GO Slim terms not predicted by the feature classifier using the IDR features at probability *p* or higher.

The recall (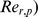 and precision (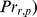 for each IDR were calculated as shown in equations (4) and (6) respectively. The overall precision at probability p was calculated as
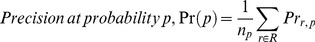
(8)where 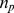 = number of IDRs that correctly predict at least one GO Slim term at probability *p* or higher and 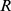 = total number of IDRs in the test set

The average recall at probability p or higher was calculated as
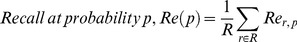
(9)


We then plotted the overall precision and recall values at various prediction probability values for each feature classifier.

Along with the complete set of intrinsically disordered proteins from Moesa et al. [9], we also separately evaluated the performance of the sequence features on three conservation-based subsets of the data (Text S1).

## Results and Discussion

### Overall Evaluation of Sequence Features

Seven sequence features were selected for evaluation of their performance using Naïve Bayes classifier to predict protein function using IDR sequence alone (Table 1). We have previously shown that chemical composition is maintained in some IDRs with poor sequence conservation. Additionally, it can also be associated with protein function hence making a good starting feature [9]. Amino acid composition of IDRs was chosen since it has been used to predict protein function at rates better than random [12]. We also used several sequence features that have been previously used for protein fold recognition but have not been explicitly evaluated in the context of proteins with intrinsic disorder. Specifically, the Dubchak features take into account the amino acid hydrophobicity, normalized van der Waals volume, predicted secondary structure, polarity and polarizability [15]. Consecutive bigram frequencies of the IDRs represent the frequencies of pairs of amino acids within the IDR. Similarly, alternate bigram frequencies represent the prevalence of pairs of amino acids separated by a single residue [17]. Finally, we include a feature that uses information based on remote homology in the form of bigram probabilities calculated from PSIBlast PSSMs of the IDRs [18].

**Figure 2:**
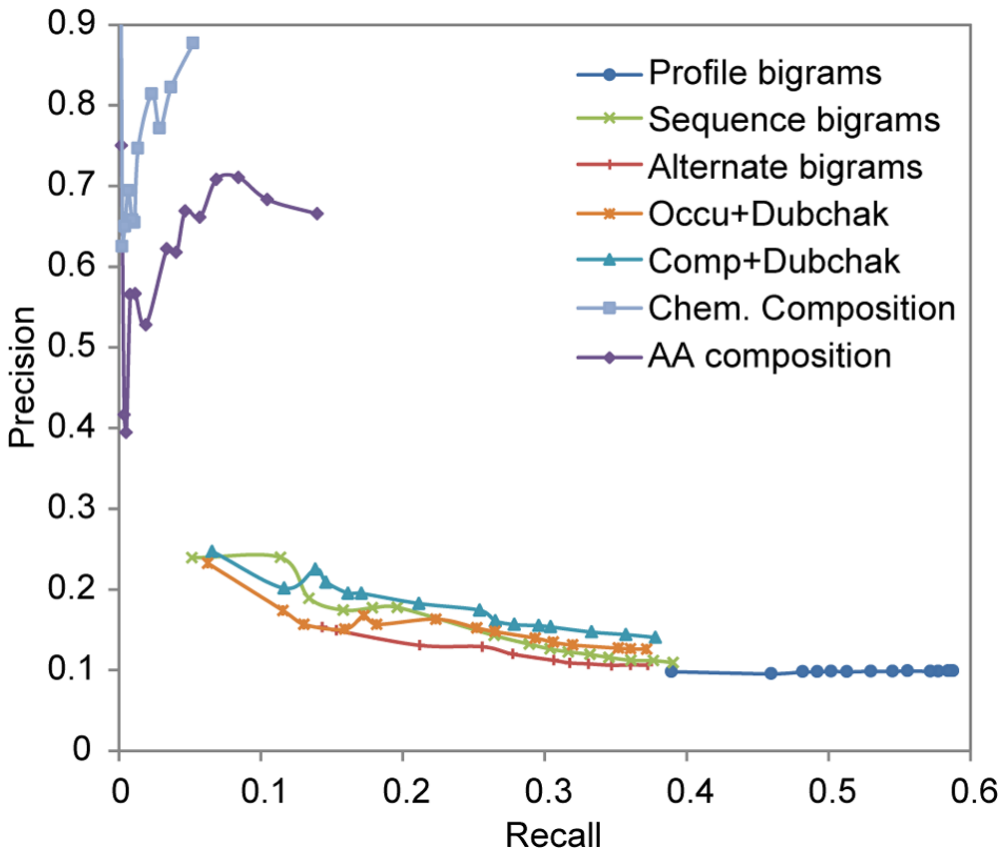
Precision-recall plots comparing the performance of the sequence feature classifiers. Precision-recall curves for the prediction of GO Slim terms by Naïve Bayes classifier using 7 sequence features of IDRs. Abbreviations used: AA – amino acid, Chem – chemical, Comp – composition, Occu – occurrence.

To evaluate each feature, independent classifiers were trained for all 130 GO Slim terms that were associated with the proteins containing the IDRs in the dataset. Naïve Bayes classifier had the highest sensitivity among a set of classifiers tested (Figures S2 and S3, Table S3). Hence we performed all further classification using the Naïve Bayes classifier. 10-fold cross validation was performed on the set of protein IDRs for each GO Slim term to evaluate the performance of each feature. Table 2 shows the average sensitivity, specificity and precision obtained for the classifiers of all GO Slim terms for each feature. We further evaluated the performance of each feature using the precision-recall curve (Figure 2).

**Table 2:** Average performance of sequence features in IDRs using 10-fold cross-validation for all 130 GO Slim terms tested.

Feature	Recall/Sensitivity	Specificity	Precision
Chemical composition	1.81	99.47	11.32
Amino acid composition	6.07	97.54	10.02
Composition+Dubchak features	30.41	80.68	8.08
Occurrence+Dubchak features	33.29	77.66	7.90
Alternate bigrams	37.04	73.52	8.50
Sequence bigrams	38.77	73.21	8.61
Profile bigrams	50.39	59.22	7.13

The results indicate that the amino acid composition and the chemical composition have the highest precision values. The Dubchak features significantly increased the true positive rate, or recall, indicating that they can capture more correctly the nature of the IDRs. Amino acid composition in combination with the Dubchak features performed slightly better than occurrence with Dubchak features in terms of precision, though both features had similar recall. The alternate and sequence bigrams showed similar levels of precision and recall to the Dubchak features. Among these, the sequence bigrams had a higher precision than the alternate bigrams. Finally, bigram probabilities obtained from PSIBlast profiles have the highest recall but at the expense of precision. This is the only sequence feature that uses sequence homology. The higher recall values of the profile-based bigram probabilities could in part be the result of partial motif identification within IDRs. Indeed, motifs have been found in human and yeast proteins within IDRs [6], [7]. However, this feature suffers from a marginal lack of coverage due to the inability of PSIBlast to create profiles for certain IDRs. The sequence features described here have been previously used to predict protein folds. It is interesting to note that features used for fold prediction perform reasonably well for IDR characterization and protein function prediction. We show here that they can also be independently used to reasonably predict protein functional terms better than existing systems using amino acid composition.

We conclude that no single sequence feature outperforms the others in terms of both precision and recall suggesting that a combination of features will be more meaningful.

### GO Slim Term Specific Evaluation of Sequence Features

In order to clarify the differences in the prediction performance of different functional terms, we calculated the Mathew’s correlation coefficient (MCC) for each GO Slim term per IDR feature (Table 3, Table S4). MCC values greater than 0 indicate a better than random performance of the classifier. All feature classifiers were able to predict GO Slim terms with MCC values greater than 0.05. Functions frequently associated with intrinsically disordered proteins such as nucleic acid binding, transcription factor activity, mRNA binding, signal transduction and histone binding had high MCC values across multiple features and showed an overall good prediction performance.

**Table 3:** MCC values for 7 IDR sequence features for the top 30 GO Slim terms predicted by at least 4 features with MCC >0.05.

GO Slimterm	Description	Chemicalcomposition	Aminoacidcomposition	Composition+Dubchak	Occurrence+Dubchak	Sequencebigrams	Alternatebigrams	Profilebigrams
GO:0005578	Proteinaceous extracellularmatrix	0.288	0.346	0.212	0.103	0.182	0.171	0.222
GO:0030198	Extracellular matrixorganization	0.302	0.210	0.121	0.131	0.220	0.204	0.121
GO:0005576	Extracellular region	0.215	0.266	0.174	0.098	0.149	0.163	0.162
GO:0006397	mRNA processing	0.178	0.189	0.139	0.146	0.176	0.152	0.044
GO:0005198	Structural molecule activity	0.171	0.203	0.113	0.133	0.126	0.117	0.102
GO:0001071	Nucleic acid bindingtranscriptionfactor activity	−0.005	0.149	0.148	0.133	0.190	0.175	0.138
GO:0005634	Nucleus	0.093	0.108	0.131	0.140	0.144	0.134	0.088
GO:0048856	Anatomical structuredevelopment	0.071	0.113	0.124	0.116	0.132	0.120	0.110
GO:0003723	RNA binding	0.077	0.113	0.116	0.117	0.118	0.095	0.102
GO:0003677	DNA binding	0.014	0.079	0.118	0.118	0.141	0.122	0.098
GO:0005783	Endoplasmic reticulum	0.169	0.137	0.070	0.044	0.084	0.096	0.072
GO:0034641	Cellular nitrogen compoundmetabolic process	0.096	0.094	0.079	0.079	0.114	0.107	0.056
GO:0009790	Embryo development	–	0.067	0.119	0.104	0.114	0.118	0.099
GO:0048646	Anatomical structureformation involved inmorphogenesis	0.040	0.079	0.096	0.074	0.102	0.100	0.085
GO:0030154	Cell differentiation	0.030	0.065	0.109	0.091	0.093	0.080	0.091
GO:0051276	Chromosome organization	0.024	0.084	0.100	0.107	0.096	0.089	0.058
GO:0042254	Ribosome biogenesis	0.034	0.067	0.113	0.106	0.084	0.079	0.060
GO:0016887	ATPase activity	0.040	0.046	0.100	0.100	0.092	0.098	0.056
GO:0005730	Nucleolus	0.038	0.064	0.104	0.096	0.074	0.068	0.071
GO:0006259	DNA metabolic process	0.013	0.065	0.105	0.095	0.099	0.081	0.040
GO:0034655	Nucleobase-containingcompound catabolic process	0.057	0.062	0.098	0.095	0.071	0.083	0.029
GO:0005654	Nucleoplasm	0.060	0.055	0.046	0.047	0.088	0.100	0.072
GO:0005694	Chromosome	0.014	0.069	0.090	0.078	0.085	0.082	0.046
GO:0051082	Unfolded protein binding	0.082	0.083	0.089	0.080	0.048	0.039	0.023
GO:0022618	Ribonucleoproteincomplex assembly	0.000	0.127	0.078	0.066	0.063	0.062	0.047
GO:0005886	Plasma membrane	–	0.033	0.055	0.085	0.096	0.102	0.068
GO:0004386	Helicase activity	0.006	0.039	0.094	0.097	0.084	0.078	0.033
GO:0007165	Signal transduction	–	0.045	0.079	0.073	0.072	0.074	0.076
GO:0007049	Cell cycle	−0.008	0.071	0.075	0.073	0.076	0.078	0.049
GO:0042393	Histone binding	−0.007	0.080	0.082	0.059	0.071	0.068	0.033

GO Slim terms related to the extracellular matrix showed the best prediction performance by all the features. Terms related to DNA and RNA binding also had high MCC values for almost all features. Though its overall sensitivity was low (1.8%), the classifier using chemical composition had the highest average MCC value across all GO Slim terms at 0.068 followed by amino acid composition (0.054). The classifiers using Dubchak features had MCC values >0.05 for the largest number of GO Slim terms (49). The Dubchak classifiers performed well for “chromosome organization” and the related “histone binding”. Additionally, composition with Dubchak features also performed well for “signal transduction”, while occurrence with Dubchak features classifier performed best for “transmembrane transporter activity” and “helicase activity”. Sequence bigrams showed a better overall performance with good MCC values for “anatomical structure development” and “cellular nitrogen compound metabolic process”. Sequence and alternate bigrams both performed well for “nuclei acid binding transcription factor activity”. In spite of having the highest average sensitivity/recall, the profile bigram classifier had intermediate MCC values for all the GO Slim terms.

These results indicate that some features are better at predicting certain GO Slim terms than other features. A classifier using combinations of these features needs to be evaluated in the future to identify the best possible combination or a distinct set of features for each GO Slim term. The low MCC values across all features and GO Slim terms (<0.5) highlights the difficulty of predicting protein function using IDR sequence alone. As has been previously shown [10], using other features with IDR sequences will improve the performance. Our study assumes that the functions of various IDRs within the same protein are independent. Taking into account the dependencies between functions associated with different IDRs present within the same protein will provide further performance improvements. However, the fact that most feature classifiers have MCC values >0 for several GO Slim terms indicates that sequence features of IDRs can add value to function prediction systems.

Some of the terms tested here are related to GO Molecular Function and Biological Process terms that showed good classification performances using location and length of disorder, specifically those related to transcription factor activity and signal transduction [10]. The GO Slim terms with good classification performance also show some overlap with the functions previously identified for the flavors of disorder based on amino acid composition [8]. Specifically DNA binding and RNA binding were also predicted correctly by our features, though metal binding was not. While our results show some overlap with previous studies, our dataset was much more comprehensive and we analyzed several sequence features independently.

## Conclusion

We identified seven sequence features in IDRs and evaluated their ability to predict functional terms in proteins. Our results show that sequence features in IDRs can add value to function prediction methods. However, all the features here show only limited success because they are confounded by the fact that GO Slim terms assigned to proteins are often based on the functions of their ordered domains. A better estimate of prediction accuracy will be achieved by associating IDRs themselves with specific functions as is done for Pfam domains or CATH domains. Furthermore, this analysis is limited to the GO terms currently available which often ignore functions specific to IDRs. Incorporation of functional terms specific to IDRs, eg. “linker region”, will also greatly improve the accuracy of function prediction. The absence of a single feature with a superior prediction performance and the varying performance of the features for different functional terms clearly demonstrate the need to combine specific features for the prediction of different functional terms. Future work will focus on performing such an analysis.

## Supporting Information

Figure S1 **Distribution of number of Proteins and number of GOSlim terms per protein.**
(DOCX)

Figure S2 **Precision-recall curves for classifiers using the 400 dimensional feature vector describing profile bigram probabilities.**
(DOCX)

Figure S3 **Precision-recall curves for the prediction of GO Slim terms by logistic regression classifier using 7 sequence features of IDRs.** Abbreviations used: AA – amino acid, Chem – chemical, Comp – composition, Occu – occurrence.
(DOCX)

Table S1 **List of proteins and their intrinsically disordered regions, along with their GO Slim terms and level of conservation.**
(XLSX)

Table S2 **The number of orthologs for each protein.**
(XLSX)

Table S3 **Performance of different classifiers using the bigram profiles feature on a selected set of IDRs.**
(XLSX)

Table S4 **MCC values for all features and all GO Slim terms tested along with the number of IDR sequences used for training.**
(XLSX)

Text S1 1) Description of the different classifiers tested, 2) Feature performance in conservation based subsets of IDRs, 3) Distribution of the number of orthologs per protein.
(DOCX)
