# Supplementary material for: Evaluation of Sequence Features from Intrinsically Disordered Regions for the Estimation of Protein Function
Source: PLoS One. 2014 Feb 24;9(2):e89890. doi: 10.1371/journal.pone.0089890 (PMC3933697; doi:10.1371/journal.pone.0089890)

## Supporting Figure S1. Distribution of number of proteins and number of GOSlim terms per protein

The graph below shows the distribution of the number of proteins with IDRs having 1 – 43 GO Slim terms. 74% of the proteins are annotated by 10 or fewer GO Slim terms.


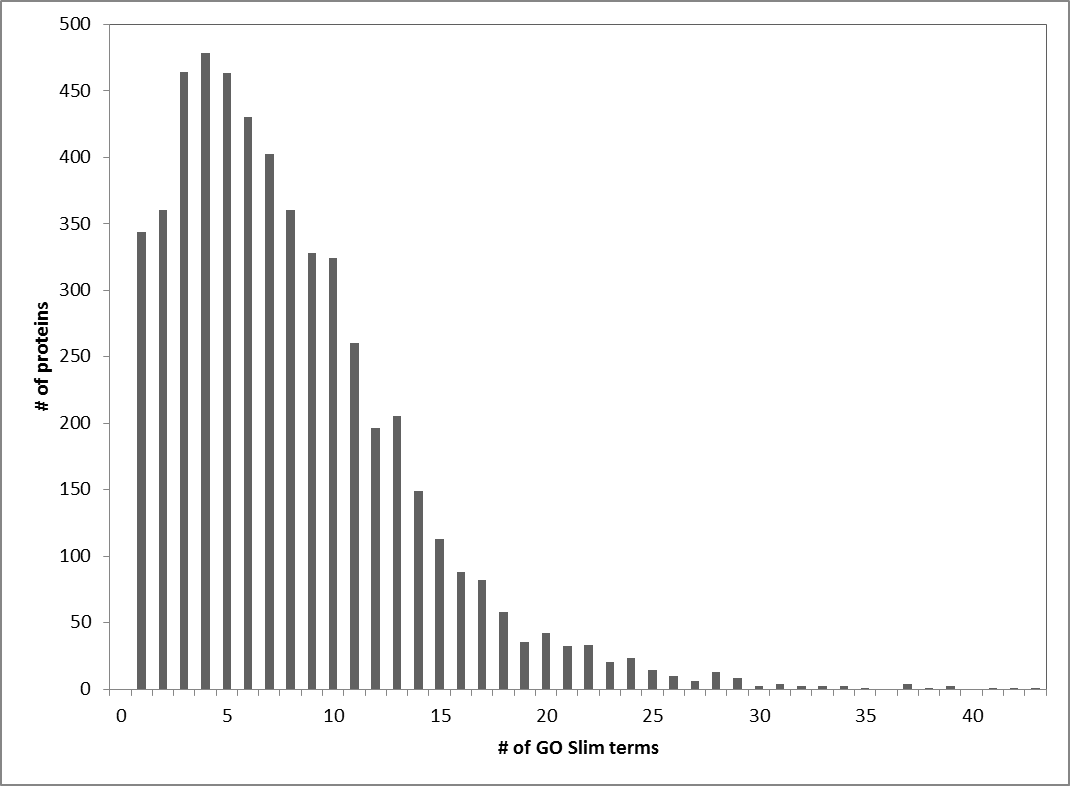

Supplement: Figure S1 — Distribution of number of Proteins and number of GOSlim terms per protein. (DOCX) [file pone.0089890.s001.docx]
