# Supplementary material for: Evaluation of Sequence Features from Intrinsically Disordered Regions for the Estimation of Protein Function
Source: PLoS One. 2014 Feb 24;9(2):e89890. doi: 10.1371/journal.pone.0089890 (PMC3933697; doi:10.1371/journal.pone.0089890)

## Supporting Figure S2. Precision-recall curves for classifiers using the 400 dimensional feature vector describing profile bigram probabilities

Naïve Bayes classifier was used for further testing due to its high recall/sensitivity than other methods.

Note: SVM was plotted as a single point since WEKA did not provide prediction probabilities < 1.


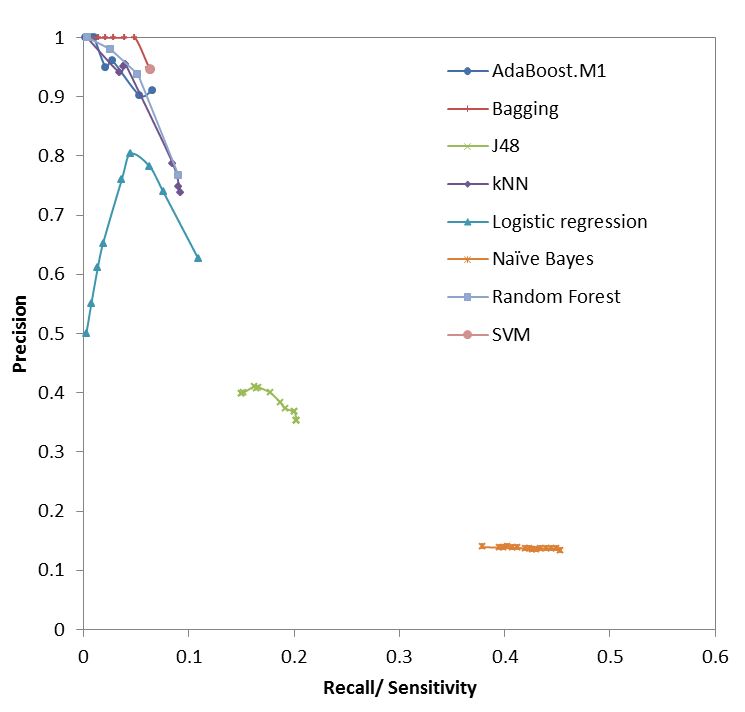

Supplement: Figure S2 — Precision-recall curves for classifiers using the 400 dimensional feature vector describing profile bigram probabilities. (DOCX) [file pone.0089890.s002.docx]
