# Supplementary material for: Evaluation of Sequence Features from Intrinsically Disordered Regions for the Estimation of Protein Function
Source: PLoS One. 2014 Feb 24;9(2):e89890. doi: 10.1371/journal.pone.0089890 (PMC3933697; doi:10.1371/journal.pone.0089890)

**Supporting Figure S3. Precision-recall curves for the prediction of GO Slim terms by logistic regression classifier using 7 sequence features of IDRs. Abbreviations used: AA – amino acid, Chem – chemical, Comp – composition, Occu – occurrence**


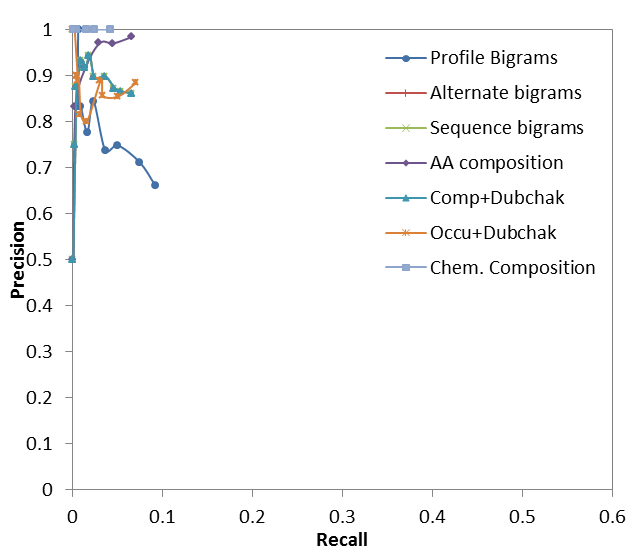

Supplement: Figure S3 — Precision-recall curves for the prediction of GO Slim terms by logistic regression classifier using 7 sequence features of IDRs. Abbreviations used: AA – amino acid, Chem – chemical, Comp – composition, Occu – occurrence. (DOCX) [file pone.0089890.s003.docx]
