# Supplementary material for: Evaluation of Sequence Features from Intrinsically Disordered Regions for the Estimation of Protein Function
Source: PLoS One. 2014 Feb 24;9(2):e89890. doi: 10.1371/journal.pone.0089890 (PMC3933697; doi:10.1371/journal.pone.0089890)
Supplement: Text S1 — 1) Description of the different classifiers tested, 2) Feature performance in conservation based subsets of IDRs, 3) Distribution of the number of orthologs per protein. (DOCX) [file pone.0089890.s008.docx]

# Supporting Information

## 1. Description of the classifiers tested

1. **Naïve Bayes**: Naive Bayes classifier is a probabilistic classifier based on applying Bayes’ theorem with strong (naive) independence assumptions. Naïve Bayes is based on presumption that the probabilities of the features are conditionally independent of each other [[1](#_ENREF_1)]. Bayesian classifier is able to simply and efficiently represent complex probability distributions.
2. **kNN**: kNN is a supervised learning approach that classifies objects based on closest samples in the feature space [[2](#_ENREF_2)]. It is a generalized form of nearest neighbor classifier. kNN rule finds the k nearest neighbors of a test sample and assigns a class label of the majority of k collected training samples.
3. **AdaBoost.M1**: AdaBoost.M1 [[3](#_ENREF_3)] is a boosting base classifier [[4](#_ENREF_4)]. It has shown high prediction accuracy for different tasks and is considered the best-of-the-shelf Meta classifier [[5](#_ENREF_5)]. Boosting is a sequential algorithm in which each new base predictor, also called weak learner [[4](#_ENREF_4)], is built based on the performance of the previously generated predictors. Firstly, a base predictor is applied to the training dataset. It updates the weights of the training data which are initially equal. In the following iterations, the training data with updated weights are given as the input to the base learner. AdaBoost.M1 is a fast and simple to program. It does not require any parameters other than the number of base learners used. It can be flexibly used with any classifier as its base leaner. AdaBoost.M1 also does not require any prior knowledge about the weak learner that it uses [[6](#_ENREF_6)].
4. **Bagging**: Bagging algorithm [[7](#_ENREF_7)] aims to build an accurate classifier. To enhance the prediction accuracy of the classification task, bagging randomly uses bootstrap samples of training dataset to train a group of classifiers and then combine the results to produce more accurate classifiers as a result of ensemble learning. Bagging focuses more on the individual accuracy of the base learners to improve the prediction performance.
5. **Logistic regression**: This is a multinomial logistic regression [[8](#_ENREF_8)] classifier which is an extension of binary logistic regression that provides a solution for the classification of more than two classes. It uses maximum likelihood estimation to evaluate the probability of class membership.
6. **J48**: J48 or C4.5 uses the fact that each feature contained in a feature vector can be employed to split the data into smaller subsets. It uses the normalized information gain (difference in entropy) to choose a feature to split the data [[9](#_ENREF_9)]. The feature with the maximum normalized information gain is applied to make the decision. The algorithm repeats on smaller lists. The decision trees generated by C4.5 are used for classification.
7. **Random Forest**: The Random Forest, proposed by [[10](#_ENREF_10)], is a straightforward modification of the Bagging classifier to enhance the diversity within the classifier ensemble [[7](#_ENREF_7)]. To overcome inefficiencies from the bagging-based approaches, two main modifications were considered in the Random Forest classifier. Firstly, Random Forest uses only decision tree as a base classifier. Secondly, each decision tree trains with the best features (among a set of randomly chosen features) to encourage diversity within the classifier ensemble.
8. **SVM**: SVM [[11](#_ENREF_11)] is considered to be a state-of-the-art machine learning and pattern classification algorithm. It has been extensively applied in classification and regression tasks. SVM aims to find a maximum margin hyperplane (MMH) to minimize classification error. In SVM, a function called the kernel K is used to project the data from the input space to a new feature space, and if this projection is non-linear it allows non-linear decision boundaries [[12](#_ENREF_12)].

## 2. Feature performance in 3 subsets of IDRs

IDRs were classified into 3 groups based on our previous study [[13](#_ENREF_13)] as:

1. Highly conserved IDRs: 6647 IDRs with high sequence conservation among aligned orthologous regions

2. IDRs with similar chemical composition across orthologs: These IDRs show relatively less sequence conservation than the highly conserved IDRs but show maintenance of chemical composition between aligned orthologous sequences. Chemical composition is determined by the fraction of different types of amino acids in the IDR and indicates the chemical nature of the IDR. This dataset consisted of 2506 IDRs.

3. Poorly conserved IDRs: 2176 IDRs that not only show poor sequence conservation but also do not show maintenance of chemical composition across orthologs.

We tested the sequence features on the 3 datasets separately to study the impact of sequence conservation on the overall performance of the classification. The difference between the accuracies of the features in the three datasets must be treated with caution. It is possible that this difference is in fact not due to the overall amount of conservation of the IDRs they contain, but the size of the training set used. The IDRs with the highest conservation have the highest sensitivity for almost all features but it is also the largest dataset affording a greater number of positive feature vectors for training.

| **Feature** | **High conservation** | | **Medium conservation** | | **Poor conservation** | |
| --- | --- | --- | --- | --- | --- | --- |
|  | **Sensitivity** | **Specificity** | **Sensitivity** | **Specificity** | **Sensitivity** | **Specificity** |
| Chemical composition | 2.37 | 99.25 | 1.07 | 99.62 | 1.45 | 99.39 |
| Amino acid composition | 7.63 | 97.22 | 6.14 | 97.06 | 2.74 | 97.67 |
| Composition + Dubchak features | 35.10 | 78.25 | 29.83 | 80.26 | 27.12 | 80.84 |
| Occurrence + Dubchak features | 37.02 | 75.51 | 30.58 | 78.61 | 27.54 | 80.38 |
| Sequence bigrams | 41.03 | 70.37 | 34.03 | 75.52 | 25.46 | 83.32 |
| Alternate bigrams | 41.44 | 70.14 | 33.38 | 75.47 | 25.03 | 82.71 |
| Profile bigrams | 49.25 | 61.96 | 40.12 | 70.22 | 35.90 | 66.42 |

## 3. Distribution of the number orthologs per protein

The distribution of the number of orthologs identified for all proteins containing the IDRs and those in each of the 3 categories based on conservation are shown in the tables below. 91% of all proteins have 4 mammalian orthologs while 72.5% proteins have at least one non-mammalian ortholog. 95.5%, 86.4% and 86.2% of proteins with IDRs showing high, medium and low conservation have at least 4 mammalian orthologs.

| Total Orthologs | High conservation IDRs | Medium conservation IDRs | Low conservation IDRs | All proteins |
| --- | --- | --- | --- | --- |
| 4 | 0.385 | 0.241 | 0.190 | 0.304 |
| 5 | 0.268 | 0.299 | 0.298 | 0.283 |
| 6 | 0.260 | 0.286 | 0.336 | 0.284 |
| 7 | 0.086 | 0.175 | 0.176 | 0.130 |

| Mammalian Orthologs | High conservation IDRs | Medium conservation IDRs | Low conservation IDRs | All proteins |
| --- | --- | --- | --- | --- |
| 1 | 0.000 | 0.000 | 0.001 | 0.000 |
| 2 | 0.001 | 0.009 | 0.011 | 0.005 |
| 3 | 0.044 | 0.127 | 0.126 | 0.084 |
| 4 | 0.955 | 0.864 | 0.862 | 0.911 |

| Non-mammalian Orthologs | High conservation IDRs | Medium conservation IDRs | Low conservation IDRs | All proteins |
| --- | --- | --- | --- | --- |
| 0 | 0.360 | 0.212 | 0.151 | 0.275 |
| 1 | 0.278 | 0.253 | 0.264 | 0.269 |
| 2 | 0.270 | 0.317 | 0.370 | 0.304 |
| 3 | 0.092 | 0.218 | 0.214 | 0.152 |

## References

1. McCallum A, Nigam K (1998) A comparison of event models for naive Bayes text classification. In AAAI-98 Workshop on Learning for Text Categorization.

2. Cover TM, Hart PE (1967) Nearest Neighbor Pattern Classification. IEEE Transactions on Information Theory 13: 21-+.

3. Freund Y, Schapire RE (1996) Experiments with a new boosting algorithm. In Machine Learning: Proceedings of the Thirteenth International Conference: 148-156.

4. Schapire RE (1990) The Strength of Weak Learnability. Machine Learning 5: 197-227.

5. Kuncheva LI, Rodriguez JJ (2007) An experimental study on rotation forest ensembles. Proceedings of the 7th international conference on Multiple classifier systems LNCS 4472: 459-468.

6. Friedman J, Hastie T, Tibshirani R (2000) Additive logistic regression: A statistical view of boosting. Annals of Statistics 28: 337-374.

7. Breiman L (1996) Bagging predictors. Machine Learning 24: 123-140.

8. Retherford RD, Choe MK (1993) Statistical models for causal analysis: John Wiley & Sons, Inc.

9. Quinlan JR (1993) C4.5: Programs for Machine Learning, Machine Learning: Morgan Kaufmann Publisher.

10. Breiman L (2001) Random forests. Machine Learning 45: 5-32.

11. Vapnik VN (1995) The nature of statistical learning theory. New York: The nature of statistical learning theory.

12. Bishop CM (2006) Pattern recognition and machine learning: Springer Science, NY.

13. Moesa HA, Wakabayashi S, Nakai K, Patil A (2012) Chemical composition is maintained in poorly conserved intrinsically disordered regions and suggests a means for their classification. Mol Biosyst 8: 3262-3273.
